# Supplementary material for: PEG10 viral aspartic protease domain is essential for the maintenance of fetal capillary structure in the mouse placenta
Source: Development. 2021 Sep 24;148(19):dev199564. doi: 10.1242/dev.199564 (PMC8497776; doi:10.1242/dev.199564)
Supplement: Supplementary information [file develop-148-199564-s1.pdf]

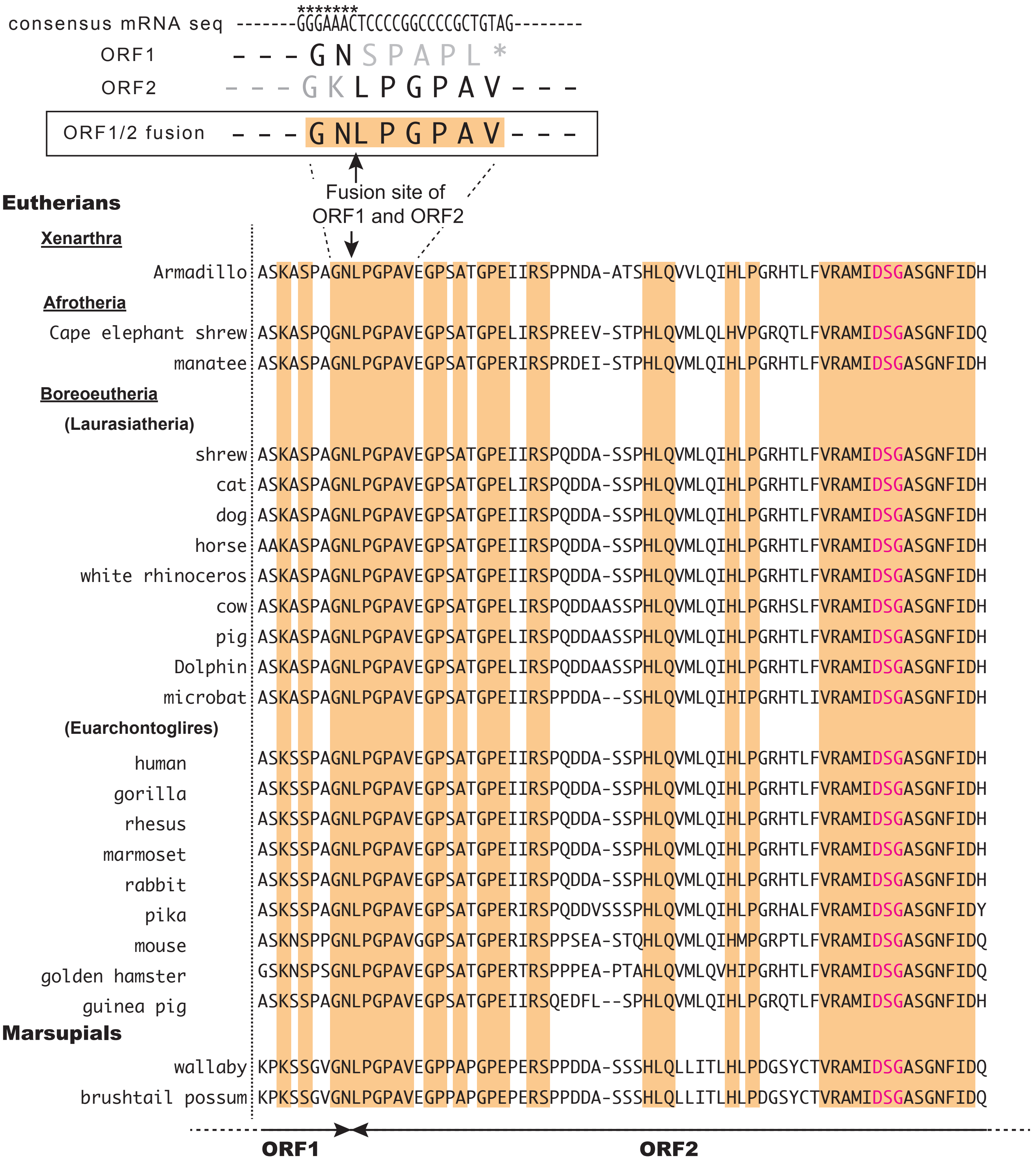

Fig. S1. The PEG10 ORF1/2 fusion protein and its DSG viral aspartic protease motif are highly conserved among all of the therian mammals.

Top: *PEG10* has a consensus slippery sequence, GGGAAAC (=XXXYYYZ) that is essential for the -1 frameshift that results in an ORF1/2 fusion protein between the ORF1 C-terminus and the head of ORF2. Bottom: Conservation of the amino acid sequence of the ORF1/2 fusion region as well as the conserved DSG protease motif (shown in red) in the ORF2 in the eutherian and marsupial species, indicating the importance of the ORF1/2 fusion protein and its protease activity.

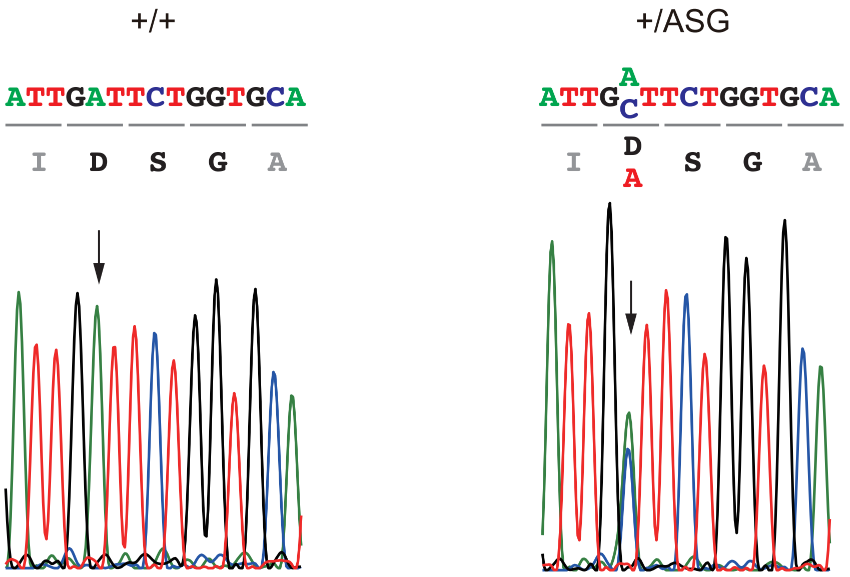

Fig. S2. Genotyping of the +/+ and +/ASG mice.
A point mutation, A to C, results in the amino acid substitution, DSG to ASG, in +/ASG mice.

Development • Supplementary information

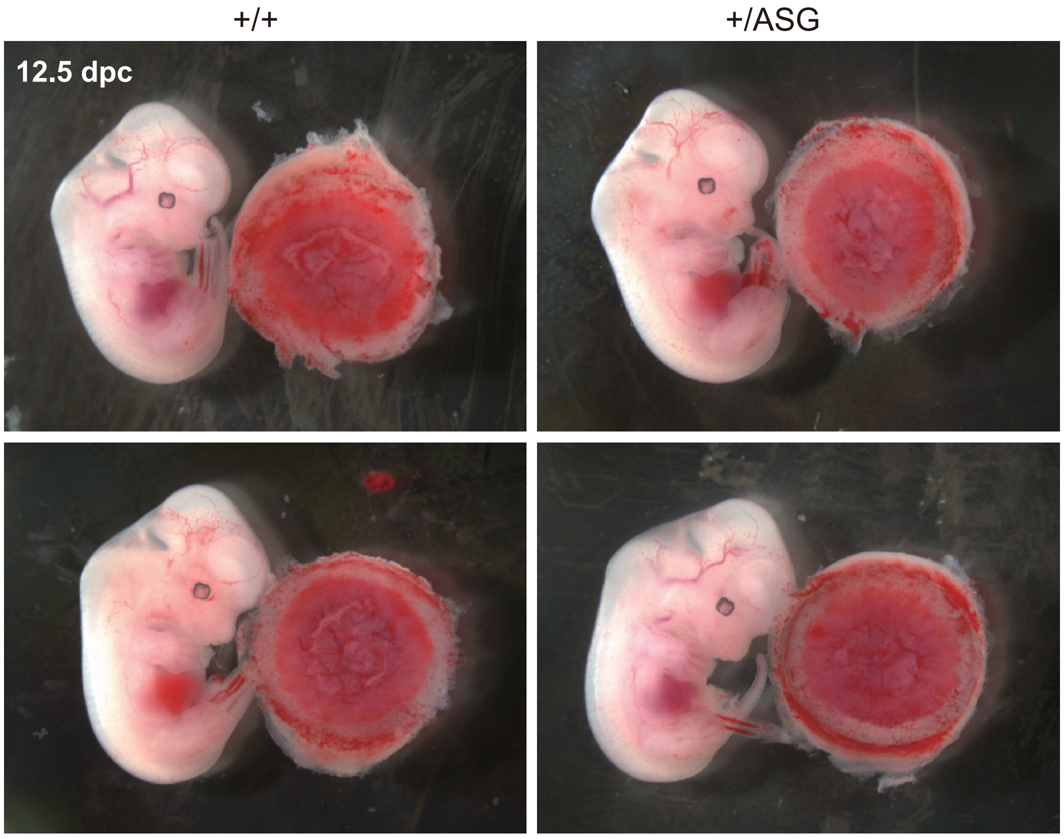

Fig. S3. Both embryos and placentas of *Peg10*-ASG are apparently normal at 12.5 dpc.+/+ (left) and +/ASG (right) embryos are shown along with their placentas at 12.5 dpc.

Development • Supplementary information

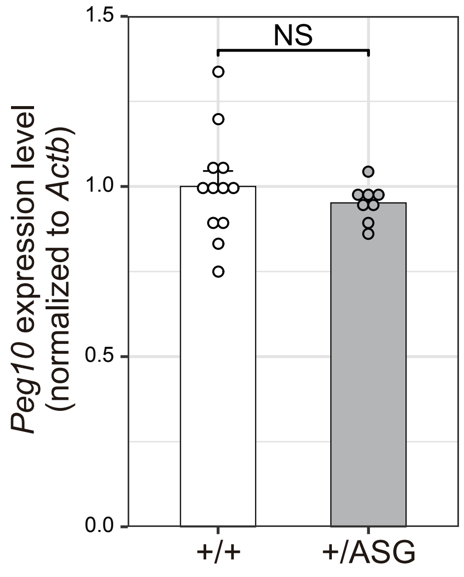

Fig. S4. *Peg10* expression level in 12.5 dpc placenta.
The *Peg10* expression level was determined by quantitative PCR analysis. The value of '1' represents the mean *Peg10* expression level in the +/+ placenta. In total 20 samples from two litters (+/+ : +/ASG = 12 : 8) were examined. The mean values of each genotype were plotted and each dot indicates the value obtained from each sample. Statistical significance was calculated using a two-tailed unpaired Student's t-test with Welch's correction. The error bars indicate the s.e.m. NS; Not significant.

Development • Supplementary information

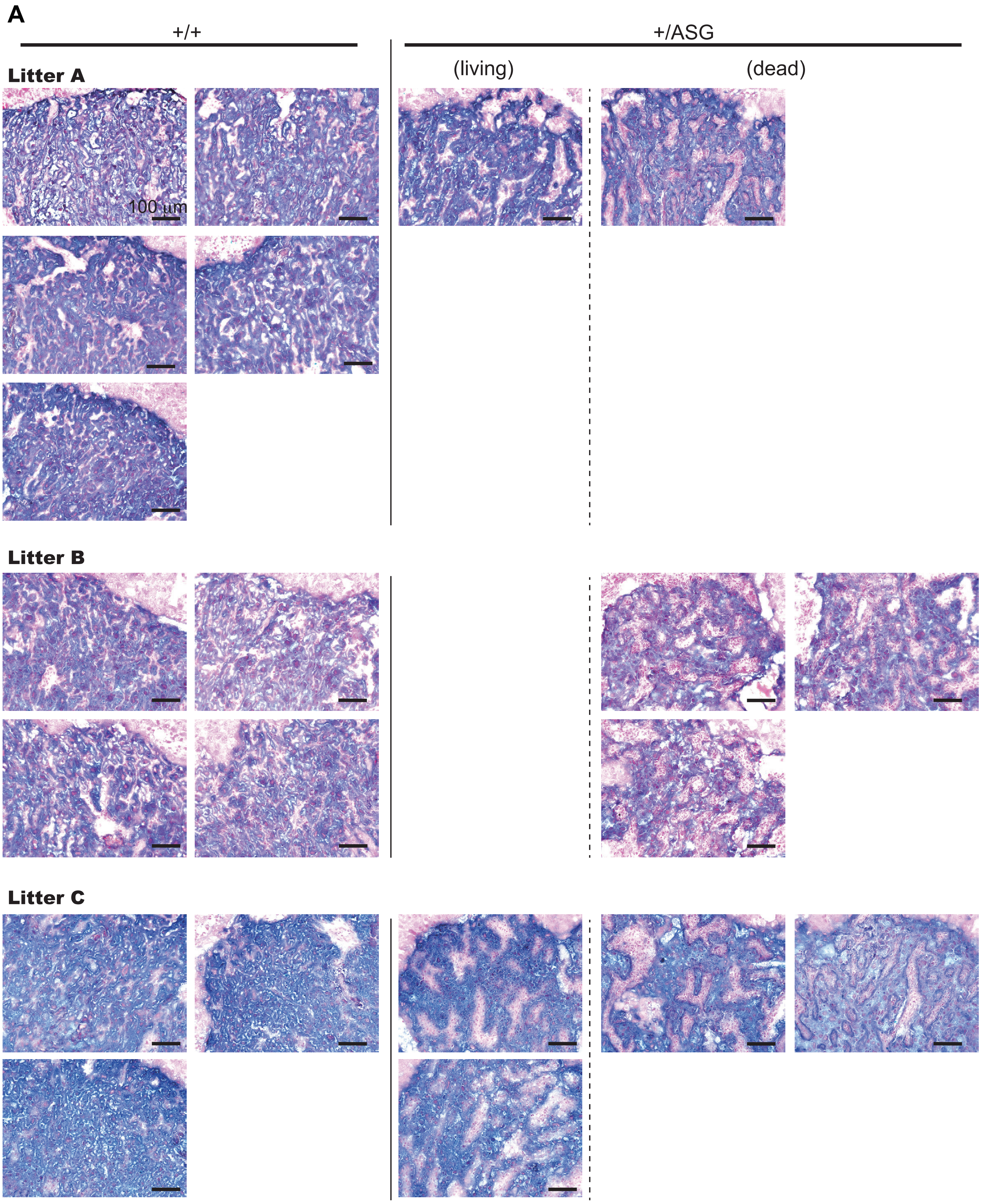

Development • Supplementary information

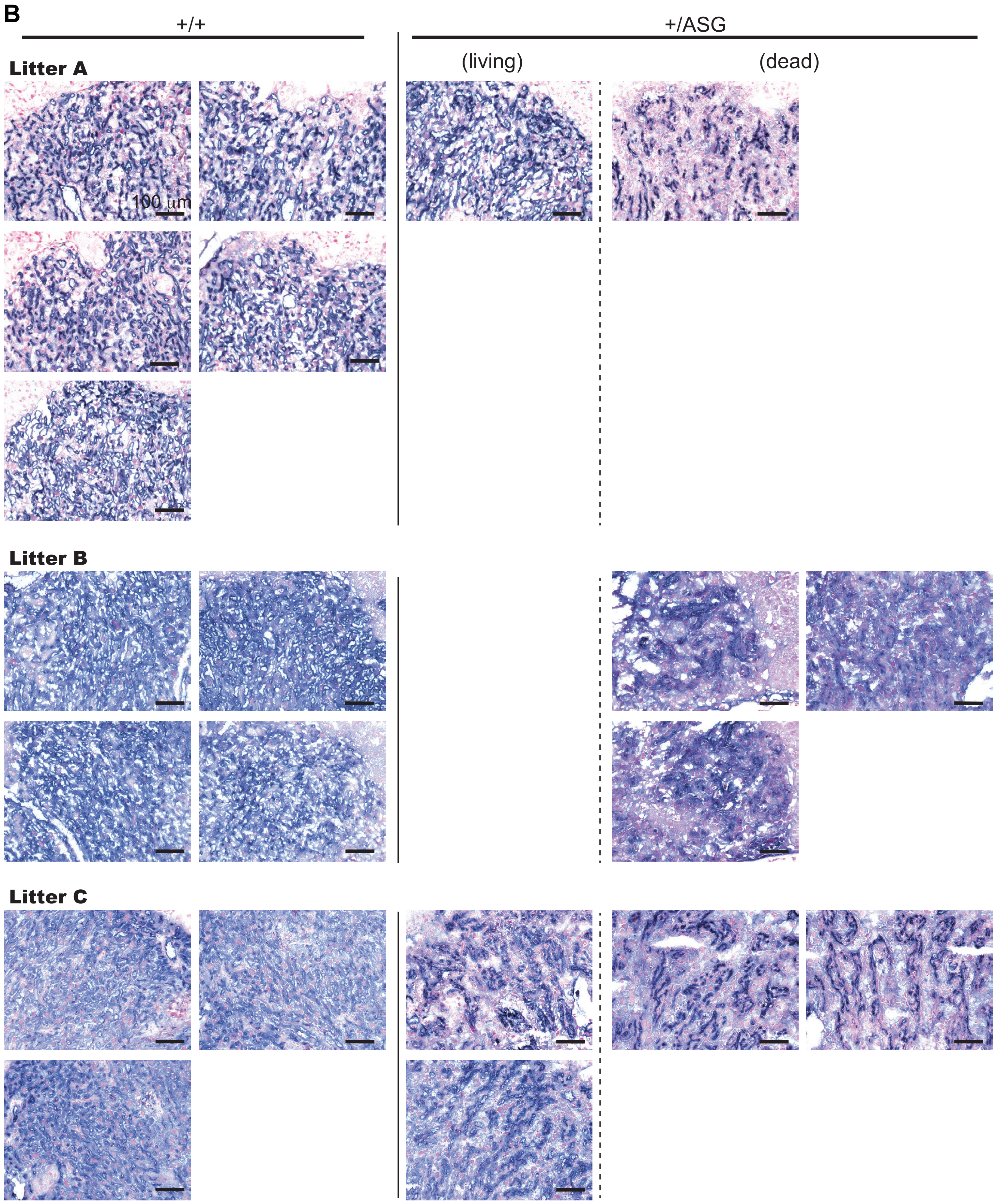

Development • Supplementary information

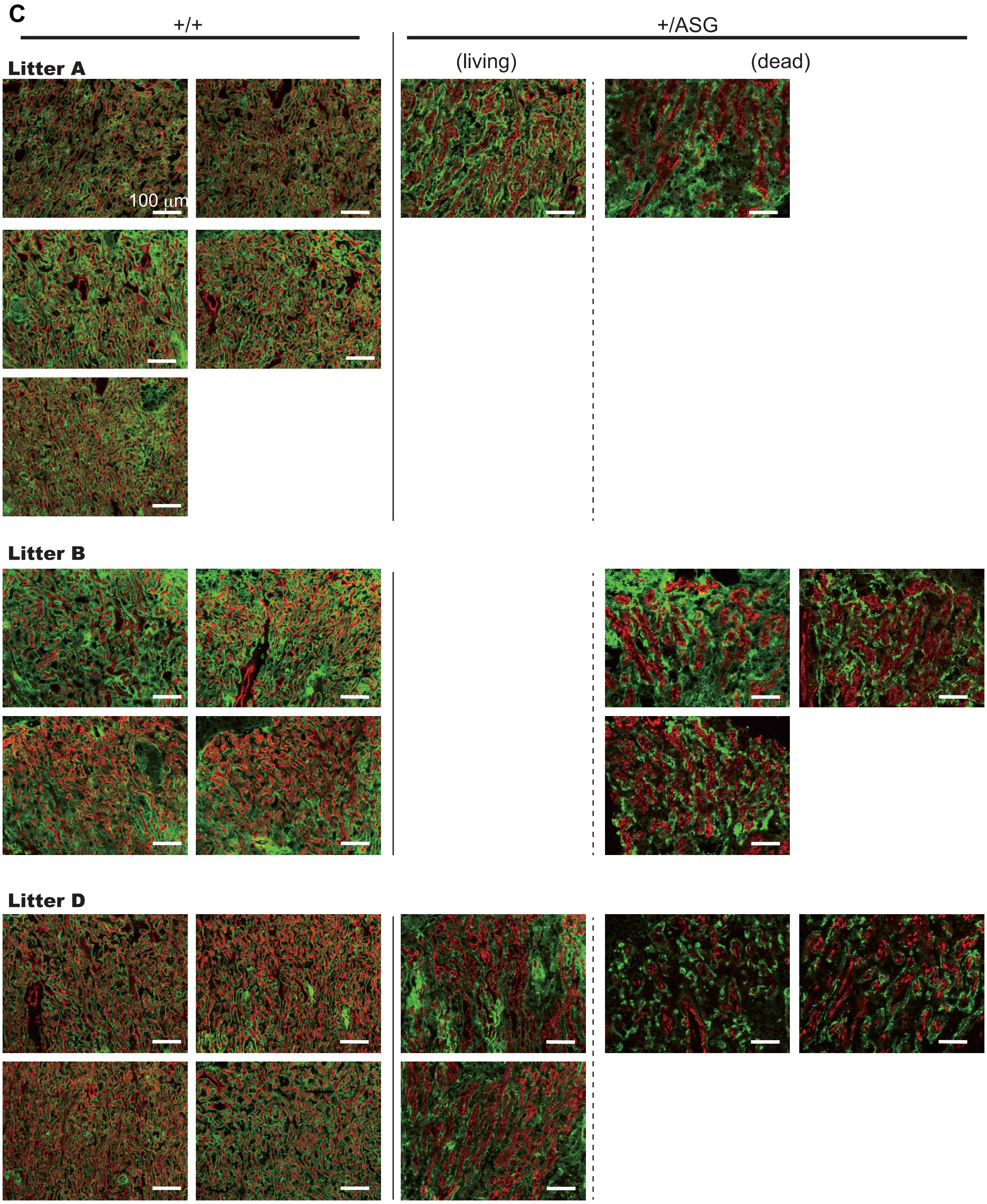

Development • Supplementary information

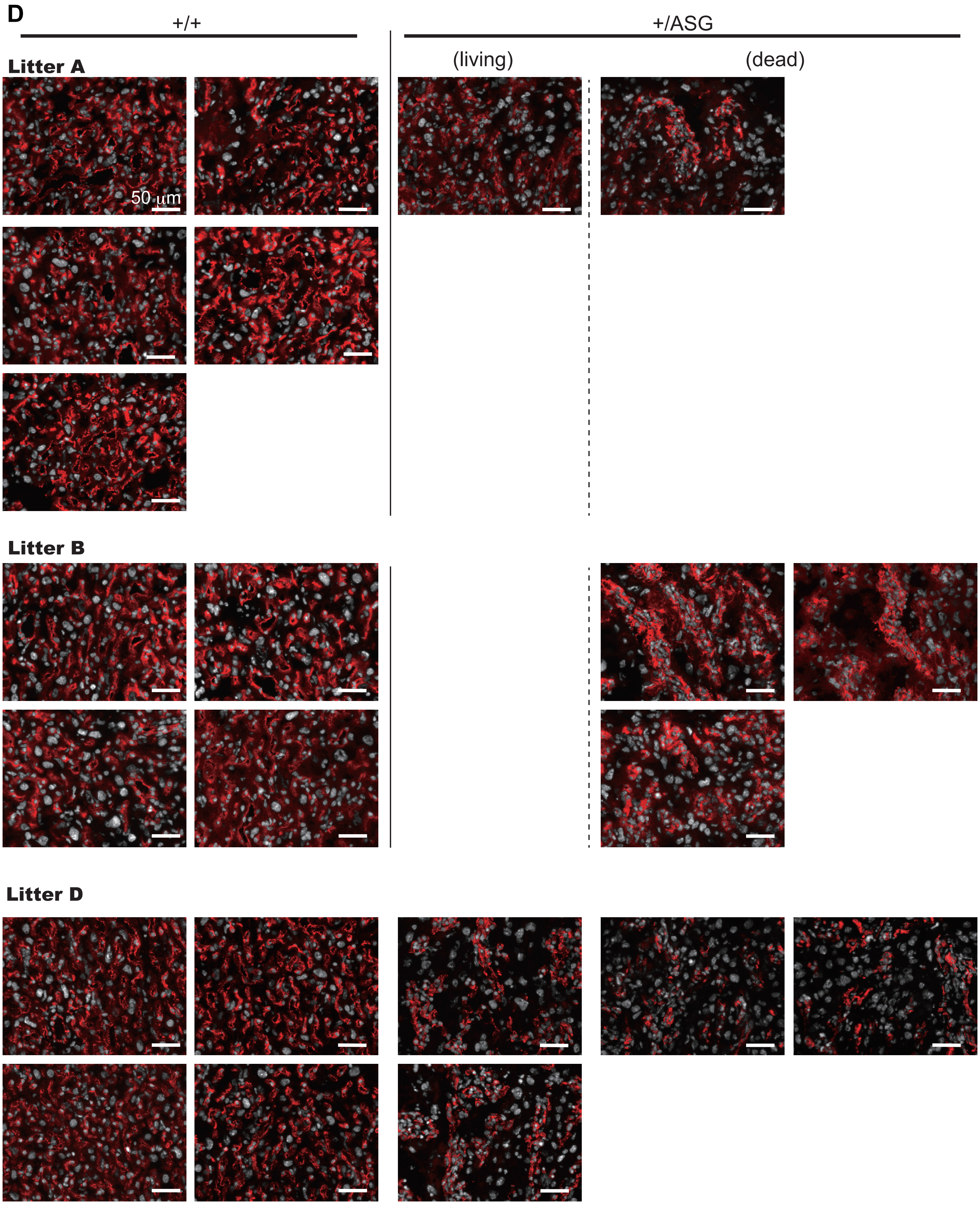

Fig. S5. Additional images related to Figure 2.
(A) Alkaline phosphatase (AP) staining and (B) immunohistochemical staining with an anti-CD31 antibody of the 18.5 dpc labyrinth layers, respectively. Nuclei were stained with Nuclear Fast Red. (C) Immunofluorescence analysis of the 18.5 dpc labyrinth layers with an anti-cytokeratin (CK) (green) and CD31 (red) antibodies. (D) Magnified images of the immunofluorescence with an anti-CD31 antibody (red) and nuclear staining with DAPI (white). For each analysis, all of the placentas recovered from three litters were examined.

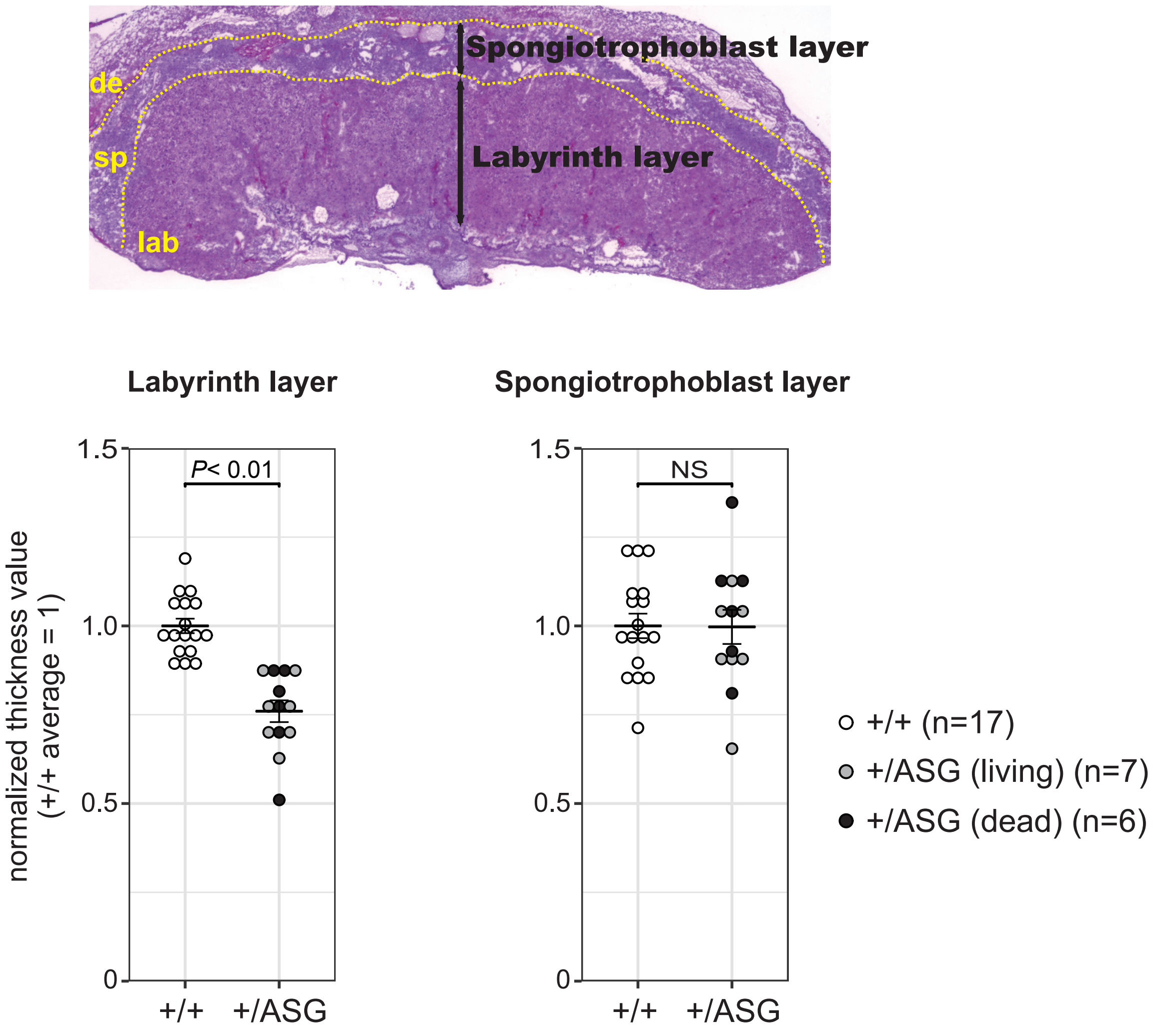

Fig. S6. Analysis of the size of labyrinth and spongiotrophoblast layers.

The thickness of the labyrinth and spongiotrophoblast layers in the +/+ and +/ASG placenta at 18.5 dpc was examined. In total, 30 placentas from four litters were used for this analysis. A value of 1 represents the mean thickness of the +/+ mice. The lines within the graphs indicate the mean values for each genotype and each dot represents the value for each placenta. Statistical significance was calculated using a two-tailed unpaired Student's t-test with Welch's correction. The error bars indicate the s.e.m. NS; Not significant. The top image is a representative image of a HE stained placenta. The dotted lines indicate the borders between the maternal decidua (de) and spongiotrophoblast (sp), and between the spongiotrophoblast (sp) and labyrinth (lab) layers.

Development • Supplementary information

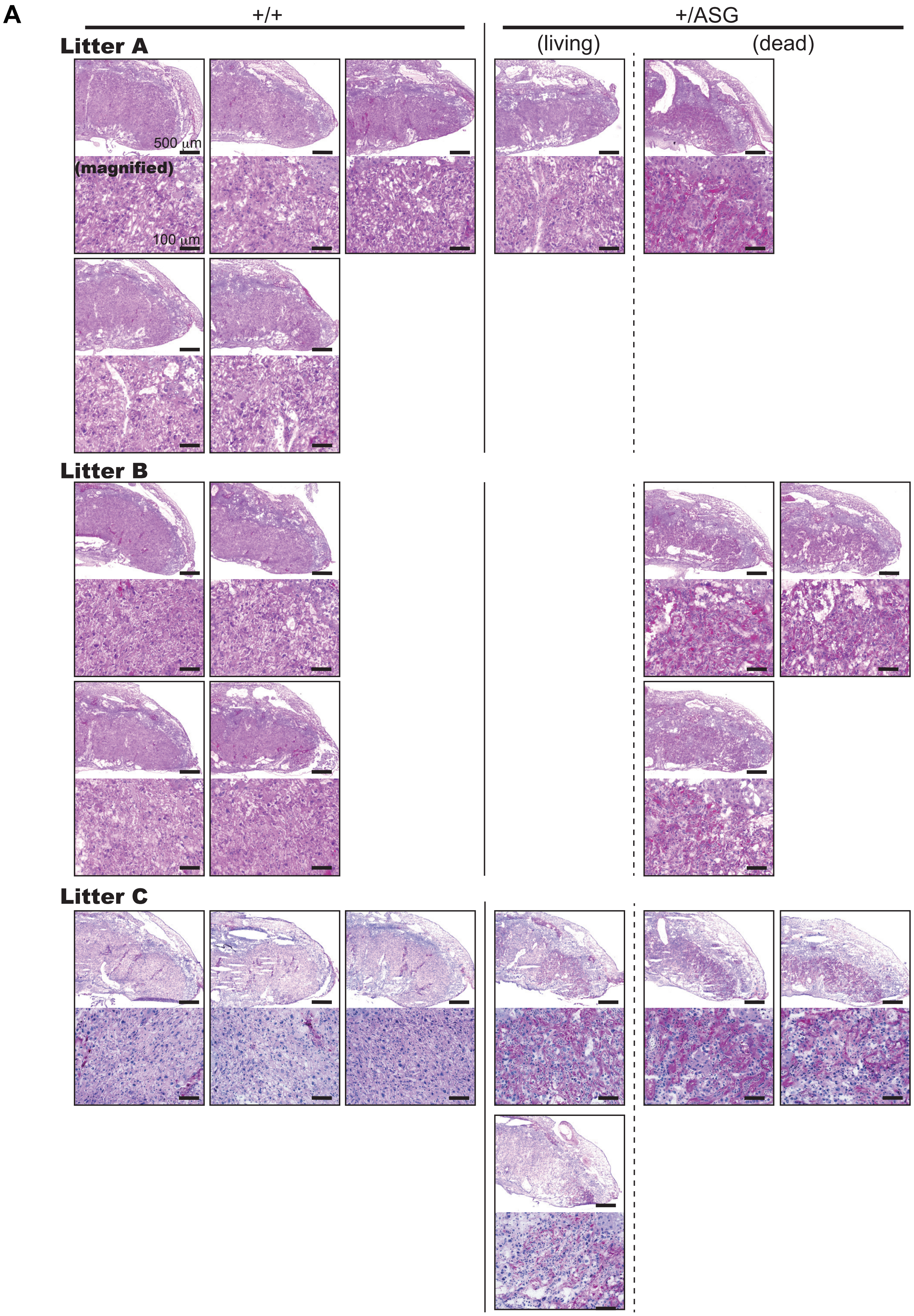

Development • Supplementary information

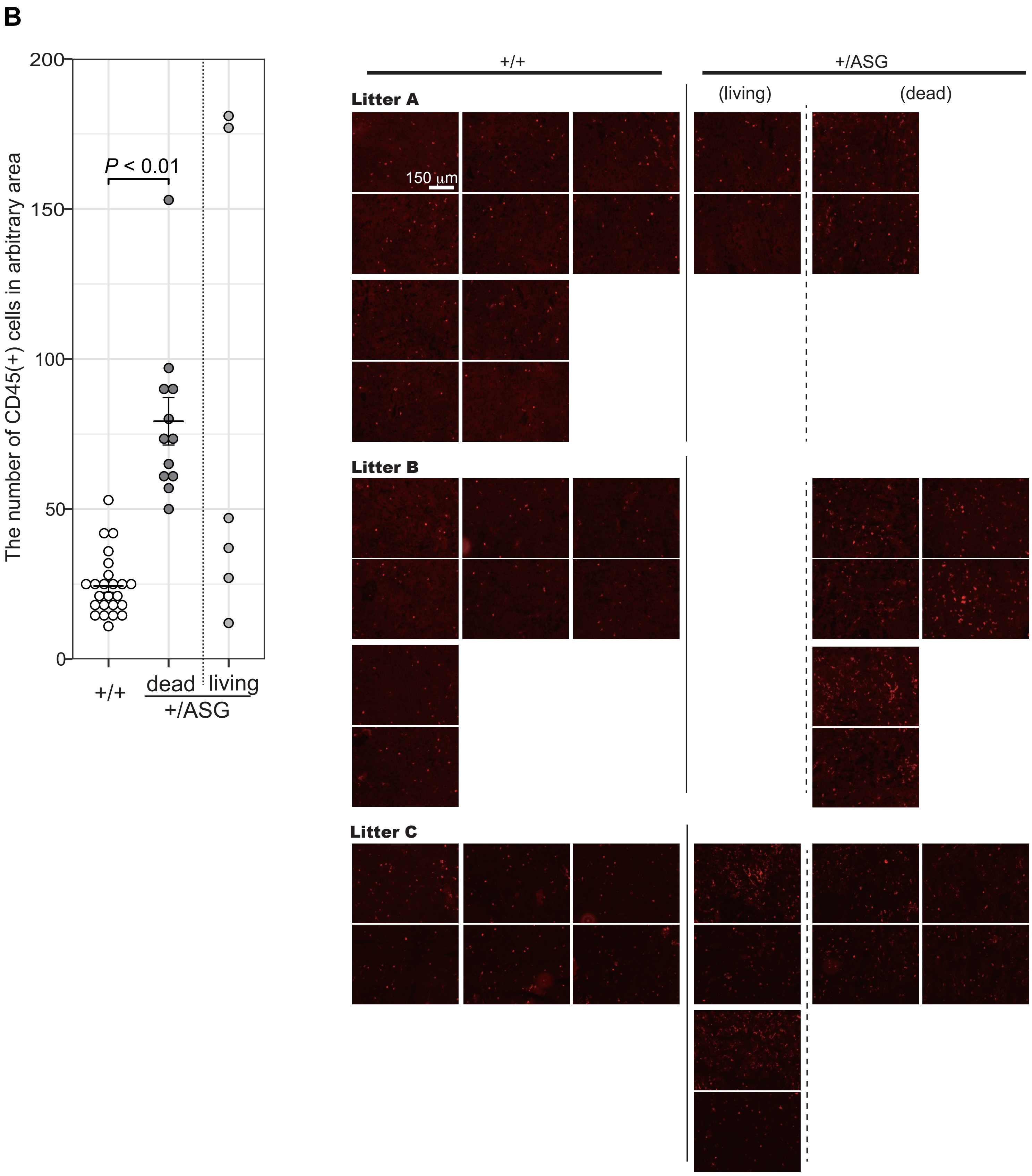

Fig. S7. Additional data related to Figures 3A and 3B.
(A) HE-stained histological sections of the 18.5 dpc placenta. (B) Comparison of the number of CD45-positive cells between +/+ and +/ASG in the 18.5 dpc placental labyrinth. Two CD45 immunofluorescence (IF) images were randomly acquired per placenta and the number of CD45-positive cells in each image was counted. The lines within the graphs indicate the mean value of each genotype and each dot represents the value acquired from each IF image. Statistical significance was calculated using a two-tailed unpaired Student's t-test with Welch's correction. The error bars indicate the s.e.m. All of the images analyzed are shown along with the plot.

Development • Supplementary information

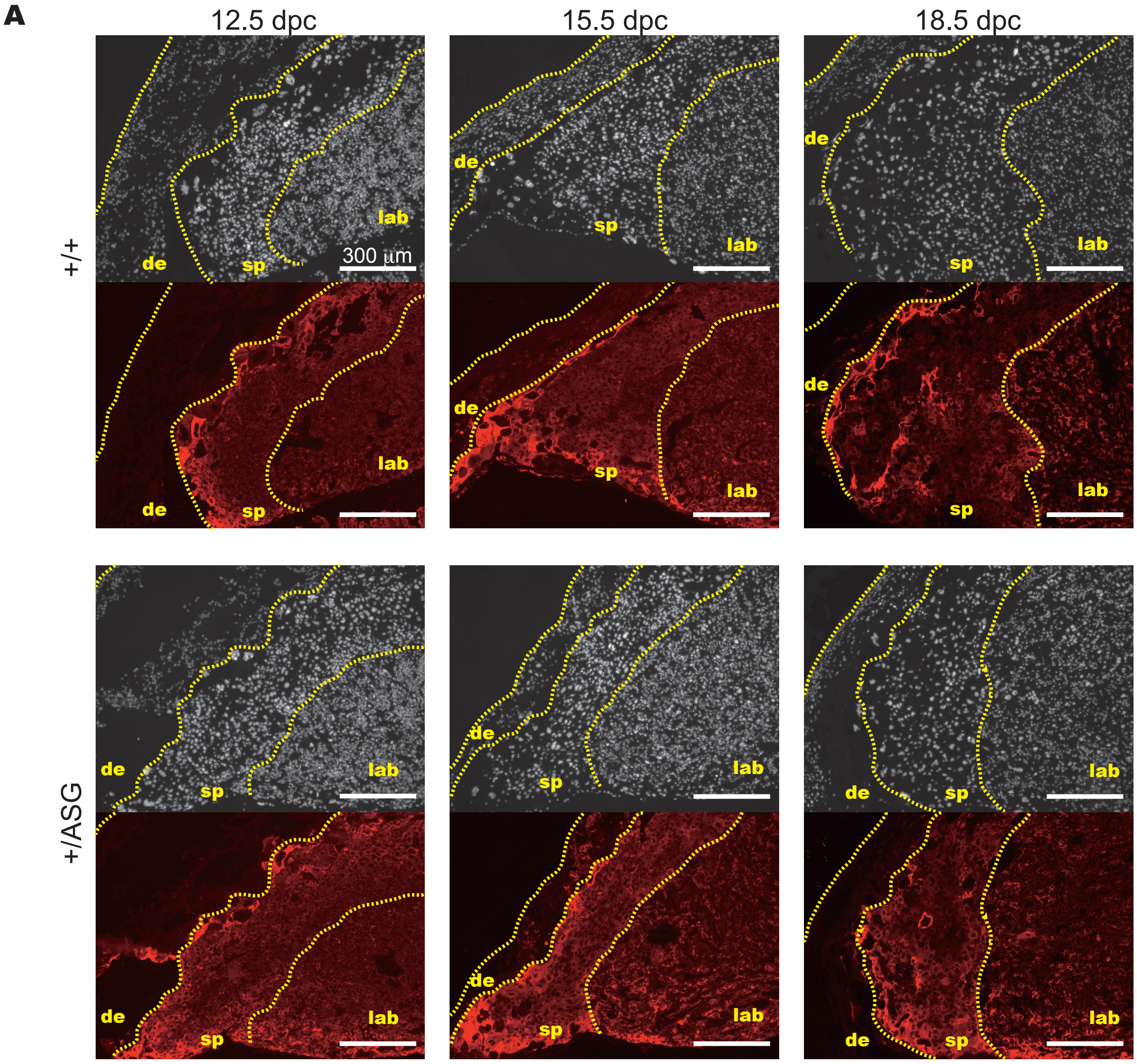

Development • Supplementary information

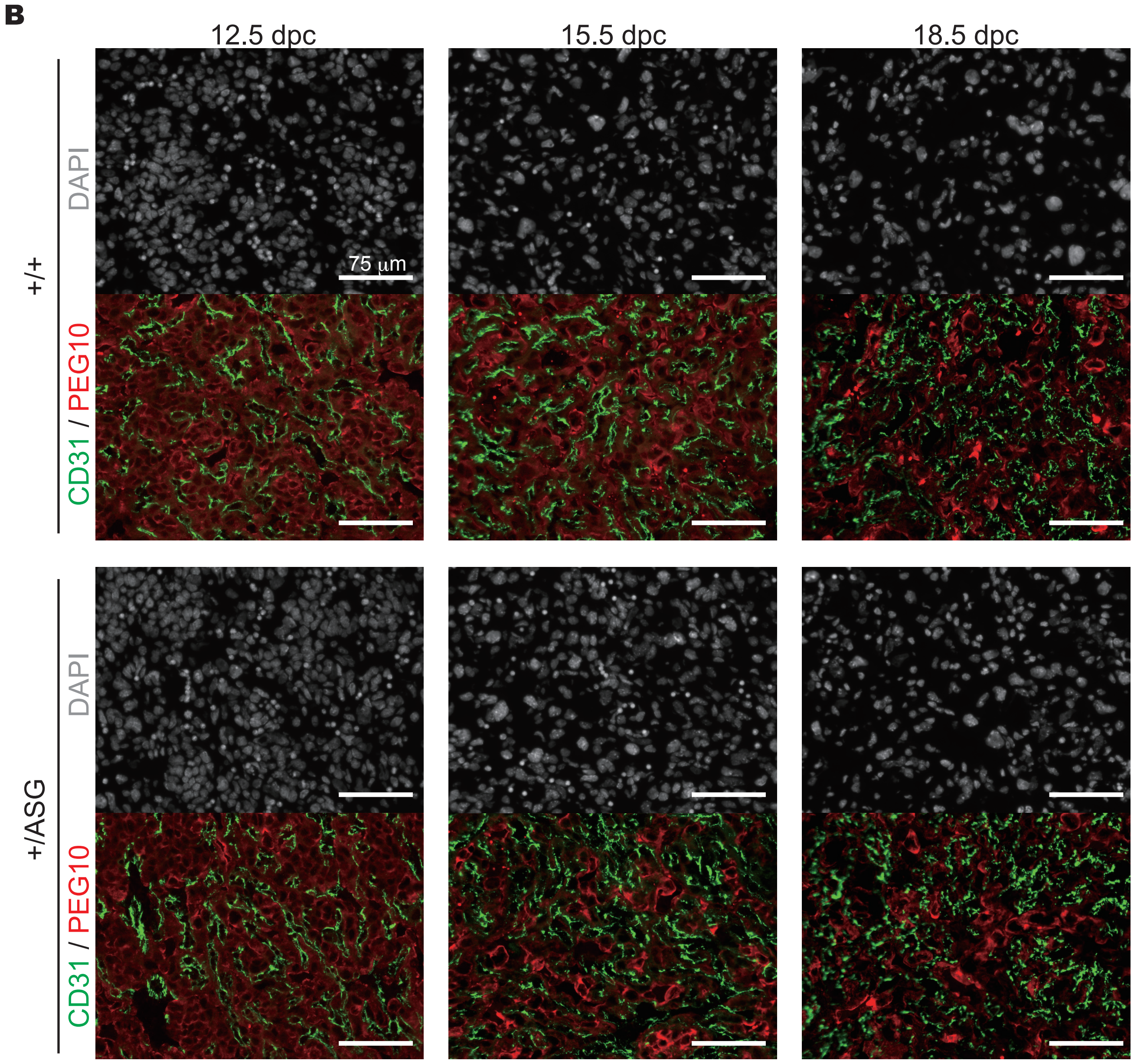

Fig. S8. PEG10 protein localization in placenta.

(A) PEG10 proteins (red) are distributed in all layers of the placenta except for the maternal decidua. The dotted lines indicate the edge of the maternal decidua, and the borders between the maternal decidua (de) and spongiotrophoblast (sp), and between the spongiotrophoblast (sp) and labyrinth (lab) layers. (B) Immunofluorescence analysis of the +/+ and +/ASG placenta with an anti-PEG10 (red) and CD31 (green) antibodies. Nuclei were stained with DAPI (white).

Development • Supplementary information

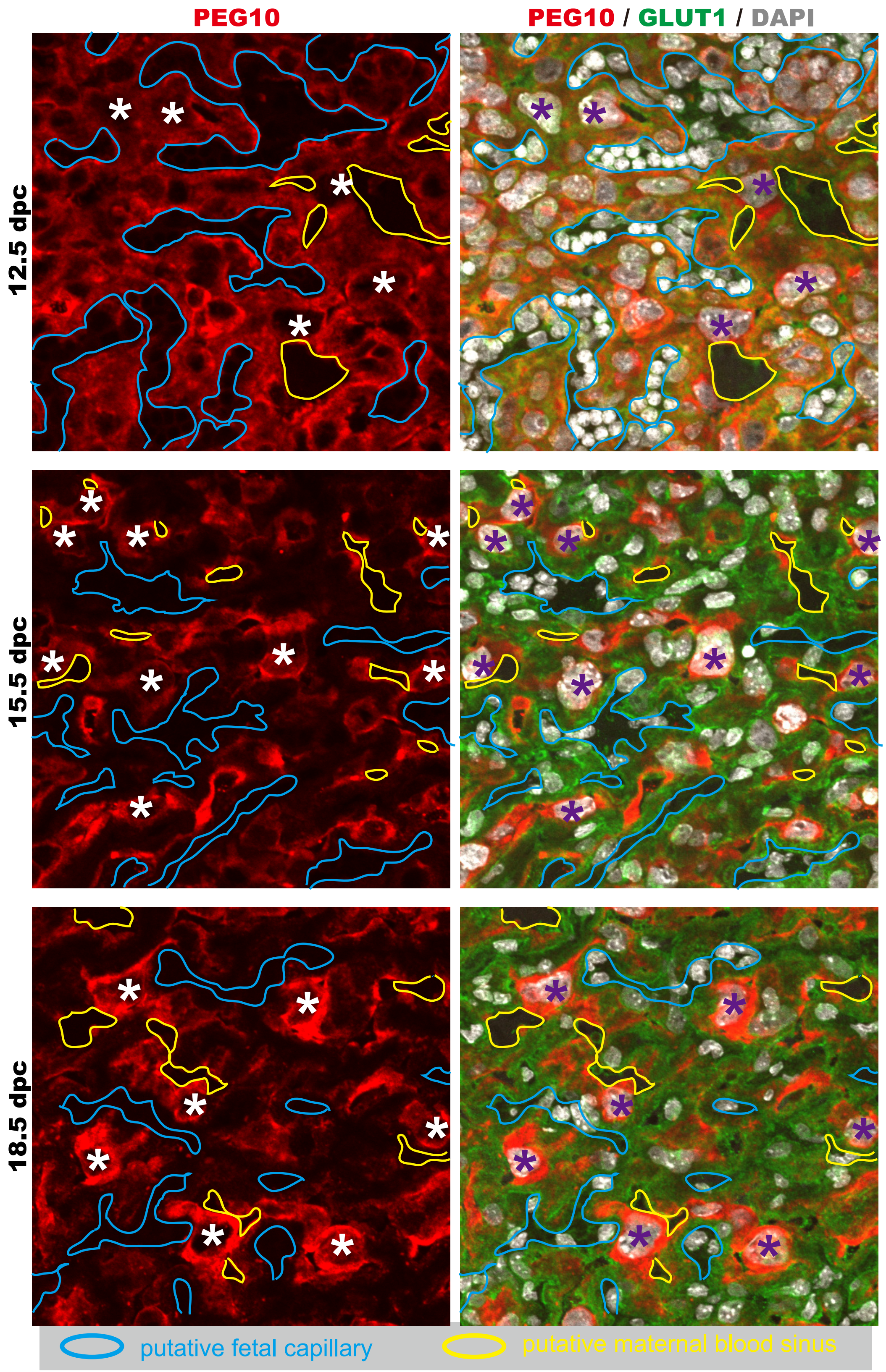

Fig. S9. Detailed analysis of PEG10 localization in placental labyrinth.
Immunofluorescence analysis of the wild-type placenta with anti-PEG10 (red) and GLUT1 (green) antibodies. Nuclei were stained with DAPI (white). GLUT1 is localized in the apical plasma membrane of the syncytiotrophoblast-I (SynT-I) facing the maternal blood sinus and basal plasma membrane of the SynT-II that faces the fetal blood capillaries. The putative fetal capillaries and maternal blood sinuses are enclosed by light blue and yellow lines, respectively. The asterisks indicate presumed s-TGCs nuclei.

Development • Supplementary information

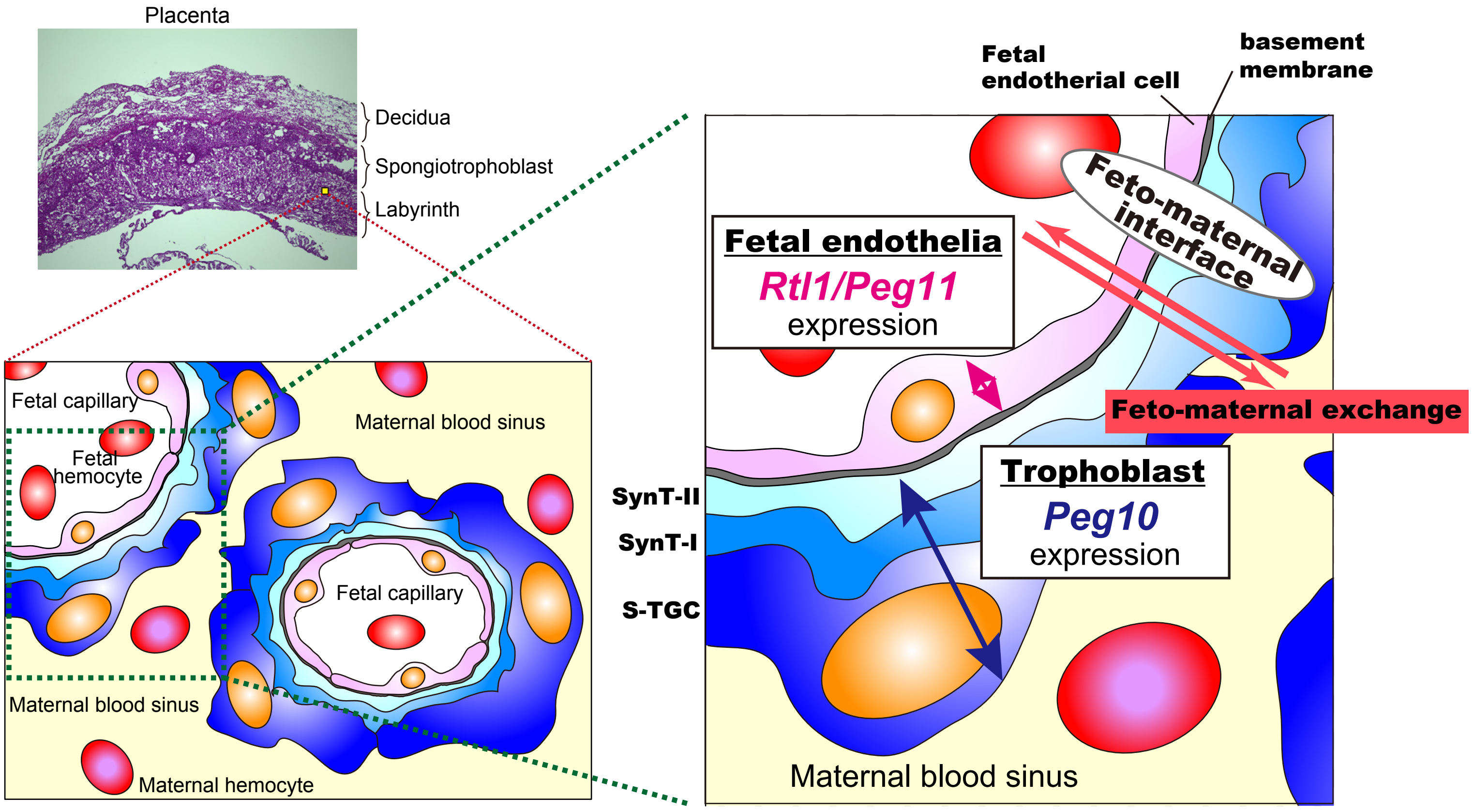

Fig. S10. A model for maintenance of fetal vascular network by *Peg10* and *Rtl1/Peg11*.
Two retrovirus-derived genes, *Peg10* and *Rtl1/Peg11*, function in trophoblast and fetal endothelial cells, respectively, acting at the feto-maternal interface to ensure normal feto-maternal exchange.

Development • Supplementary information
