# Supplementary material for: PEG10 viral aspartic protease domain is essential for the maintenance of fetal capillary structure in the mouse placenta
Source: Development. 2021 Sep 24;148(19):dev199564. doi: 10.1242/dev.199564 (PMC8497776; doi:10.1242/dev.199564)
Supplement: Reviewer comments [file develop-148-199564_review_history.pdf]

PEG10 viral aspartic protease domain is essential for the maintenance of fetal capillary structure in the mouse placenta

Hirosuke Shiura, Ryuichi Ono, Saori Tachibana, Takashi Kohda, Tomoko Kaneko-Ishino and Fumitoshi Ishino

DOI: 10.1242/dev.199564

Editor: Haruhiko Koseki

Review timeline

Original submission:	2 March 2021
Editorial decision:	21 May 2021
First revision received:	27 July 2021
Accepted:	16 August 2021

Original submission

<u>First decision letter</u>

MS ID#: DEVELOP/2021/199564

MS TITLE: PEG10 viral aspartic protease domain is essential for the maintenance of fetal capillary structure in the mouse placenta

AUTHORS: Hirosuke Shiura, Ryuichi Ono, Saori Tachibana, Takashi Kohda, Tomoko Kaneko-Ishino, and Fumitoshi Ishino

I have now received all the referees' reports on the above manuscript, and have reached a decision. The referees' comments are appended below, or you can access them online: please go to BenchPress and click on the 'Manuscripts with Decisions' queue in the Author Area.

As you will see, the referees express considerable interest in your work, but have some significant criticisms and recommend a substantial revision of your manuscript before we can consider publication. If you are able to revise the manuscript along the lines suggested, which may involve further experiments, I will be happy receive a revised version of the manuscript. Your revised paper will be re-reviewed by one or more of the original referees, and acceptance of your manuscript will depend on your addressing satisfactorily the reviewers' major concerns. Please also note that Development will normally permit only one round of major revision.

We are aware that you may be experiencing disruption to the normal running of your lab that make experimental revisions challenging. If it would be helpful, we encourage you to contact us to discuss your revision in greater detail. Please send us a point-by-point response indicating where you are able to address concerns raised (either experimentally or by changes to the text) and where you will not be able to do so within the normal timeframe of a revision. We will then provide further guidance. Please also note that we are happy to extend revision timeframes as necessary.

Please attend to all of the reviewers' comments and ensure that you clearly highlight all changes made in the revised manuscript. Please avoid using 'Tracked changes' in Word files as these are lost in PDF conversion. I should be grateful if you would also provide a point-by-point response detailing how you have dealt with the points raised by the reviewers in the 'Response to Reviewers' box. If you do not agree with any of their criticisms or suggestions please explain clearly why this is so.

© 2021. Published by The Company of Biologists under the terms of the Creative Commons Attribution License (https://creativecommons.org/licenses/by/4.0/).

Reviewer 1

Advance summary and potential significance to field

This interesting paper examines the functions of the imprinted gene PEG10 during placentation. It was known that PEG10 is essential for placentation early in pregnancy, but this study highlights additional action in the mature placenta, especially in the trophoblastic cell layers surrounding the fetal capillary endothelial cells controlling fetal and placental growth after mid-gestation. Severe defects occurred in the fetal vasculature of the placenta. The authors conducted a careful analysis of the role of PEG10 using a mutation that they generated in the highly conserved aspartic protease motif (ASG) in the viral POL-like region. The mutant mice did not have early embryonic lethality normally associated with Peg10 KO mice. Instead, only 14% of pups of the mutants survived to weaning, and 40% fetuses of the ASG mice were dead at d18.5. Surviving pups were small. They provide convincing evidence that PEG10 is essential for maintenance of fetal capillary epithelial cells and its importance for the evolution of viviparity throughout mammalian evolution.

Comments for the author

Is there any evidence for the effects on the progeny of the ASG mice? Were they fertile? The authors speculate that the trophoblast cells in fetal capillaries might have some defence mechanism to protect the endothelial cells, but the possible mechanism is unclear, or may involve a related imprinted retrotransposon gene Rtl1. It would be more convincing if this argument was fleshed out. It would also be interesting to hear the authors views on how PEG10 might function in species with epithelia-chorial or zonary placentas, as distinct from he hemochorial ones.
What do the dotted lines signify in Figure 3?

Reviewer 2

Advance summary and potential significance to field

In this manuscript Shiura et al., investigate the role of the PEG10 viral aspartic protease domain by using CRISPR-cas9 mediated genome engineering in vivo and show, using subsequent analyses on embryonic and placental growth and function, that these modifications are not embryonic lethal unlike Peg10 null embryos as previously shown. Peg10-ASG mice appear to have later defects in fetal vasculature which the authors suggest causes perinatal lethality. These results shedding light on the PEG10 viral aspartic protease domain and the parallels drawn with Rtl1 are intriguing although I have some concerns about the data presented in its current format.

Comments for the author

Major points:
1. Throughout the manuscript, all defects should be robustly quantified where possible. At present, only one representative image is shown per experiment in Fig. 2 and 3. The phenotype does appear to be quite striking from the images shown but it is currently unclear how representative these are or if there is a spectrum of these defects. For example, is the vastly increased number of CD45 positive leukocytes detected in Fig.3B consistent between +/ASG placentas from different litters? This should be addressed throughout the manuscript because at present I feel the study is descriptive and not as robust as it could be. The appropriate statistics should be used for each analysis. The number of litters, embryos and placentas analysed for each experiment should also be mentioned.

2. The authors should include confirmation that the CRISPR mutation was present as expected in the Peg10-ASG line such as Sanger sequencing of the genomic region etc. Was Peg10 gene expression disrupted or reduced?

3. From the numbers given in Table 1 (e.g 14% mutant pups versus the expected 50% at weaning) the authors should clarify the chi squared ratios are indeed significantly deviating from expected in the text or table legend for each stage. The authors should also highlight how many

© 2021. Published by The Company of Biologists under the terms of the Creative Commons Attribution License (https://creativecommons.org/licenses/by/4.0/).

litters were used for each experiment. At 12.5 dpc, the numbers of +/+ versus +/ASG embryos (25 versus 16 respectively) was similar to that at 18.5 dpc (28 versus 16 respectively) where the authors suggest the largest effect is taking place, albeit not with the same death rate as at 18.5 dpc. What might be the reason for the low number of +/ASG embryos at 12.5dpc? These numbers suggest something may indeed be occurring at 12.5 dpc, despite their comment that the mutant embryos and placentas exhibited an apparently normal appearance at 12.5 dpc (Fig. S2). The +/ASG placentas already weigh significantly less at this timepoint.

4. Is PEG10 protein localisation altered in ASG placentas? Currently staining is only shown for wild type embryos.

5. Further discussion about the potential mechanism of the PEG10 protease domain role in placental development should be included.

Minor points:
There is a spelling mistake ‘CRIPSR-Cas9’ in the first paragraph of the results. Should read CRISPR-Cas9.
Fig.3a It may be useful to highlight the labyrinth, junctional zone, decidua regions on the images. Do these regions differ in size?
Fig.3A. Highlight enlarged trophoblast nuclei as mentioned in text.
Fig.3A, 3B All analyses should be quantified in some capacity as mentioned in major point 1 Fig. S3. It might be useful to label cells of interest like the giant sinusoidal trophoblast giant cells etc. on the images.
Spelling mistake - VCTASHIELD-Hardset

First revision

Author response to reviewers' comments

Reply to the Editor
Following the reviewers’ comments and suggestions, we have made a major revision of this manuscript. We believe that these changes address all of the reviewers’ concerns and that the revised version of manuscript satisfies the criteria for publication in *Development*. Our point-by-point responses to the reviewers are listed below.

Response to reviewers’ comments
Thank you very much for reviewing our manuscript (Manuscript ID#: DEVELOP/2021/199564) entitled “PEG10 viral aspartic protease domain is essential for the maintenance of fetal capillary structure in the mouse placenta”. We greatly appreciate the helpful comments from the reviewers.

Reviewer 1 Advance Summary and Potential Significance to Field...

This interesting paper examines the functions of the imprinted gene PEG10 during placentation. It was known that PEG10 is essential for placentation early in pregnancy, but this study highlights additional action in the mature placenta, especially in the trophoblastic cell layers surrounding the fetal capillary endothelial cells controlling fetal and placental growth after mid-gestation. Severe defects occurred in the fetal vasculature of the placenta. The authors conducted a careful analysis of the role of PEG10 using a mutation that they generated in the highly conserved aspartic protease motif (ASG) in the viral POL-like region. The mutant mice did not have early embryonic lethality normally associated with Peg10 KO mice. Instead, only 14% of pups of the mutants survived to weaning, and 40% fetuses of the ASG mice were dead at d18.5. Surviving pups were small. They provide convincing evidence that PEG10 is essential for maintenance of fetal capillary epithelial cells and its importance for the evolution of viviparity throughout mammalian evolution.

© 2021. Published by The Company of Biologists under the terms of the Creative Commons Attribution License (https://creativecommons.org/licenses/by/4.0/).

Reviewer 1 Comments for the Author...

Is there any evidence for the effects on the progeny of the ASG mice? Were they fertile? The authors speculate that the trophoblast cells in fetal capillaries might have some defence mechanism to protect the endothelial cells, but the possible mechanism is unclear, or may involve a related imprinted retrotransposon gene Rtl1. It would be more convincing if this argument was fleshed out. It would also be interesting to hear the authors views on how PEG10 might function in species with epithelia-chorial or zonary placentas, as distinct from the hemochorial ones.
What do the dotted lines signify in Figure 3?
->
Response
We are glad that reviewer 1 appreciates our work and have addressed the comments in the point- by-point response provided below.

・Is there any evidence for the effects on the progeny of the ASG mice? Were they fertile?
->
Response
As described in the text, a small number of *Peg10*-ASG mice grew to be adults and had a tendency to have a lower weight than +/+ mice. We confirmed that such mutant mice, both males and females, were fertile and apparently normal, except for the small size phenotype. However, due to the limitation of the number of adult mutants, we were unable to perform a detailed phenotypic analysis. Therefore, we consider such an analysis to be a subject for the future.

・The authors speculate that the trophoblast cells in fetal capillaries might have some defence mechanism to protect the endothelial cells, but the possible mechanism is unclear, or may involve a related imprinted retrotransposon gene Rtl1. It would be more convincing if this argument was fleshed out.
・It would also be interesting to hear the authors views on how PEG10 might function in species with epithelia-chorial or zonary placentas, as distinct from the hemochorial ones.
->
Response
At this time, the defense mechanism remains unknown. We speculate that its involvement in the immune tolerance between mother and embryos is a possibility, but, elucidation of the detailed mechanism is a challenge for future work. We have added this in the discussion in addition to presumable *PEG10* functions in the species with an endotheliochorial (zonary) or epitheliochorial placenta as follows:

Line 207:
Then how do *PEG10* and *RTL1* act as guardians of the feto-maternal interface? One possibility is that PEG10 protease activity in trophoblast cells contributes to the immune tolerance of the mother to embryo antigens: for instance, the PEG10 protease may induce an aggressiveness of trophoblasts toward maternal immune cells in order to protect the fetal capillaries. Under such a scenario, RTL1 would play a defensive role in the fetal capillary endothelial cells against a collateral attack by trophoblast cells. The cooperation between PEG10 and RTL1 would thus provide the essential architecture for the feto-maternal interface in the placenta. The trophoblast cells invariably localize between the fetal capillary endothelial cells and the maternal tissues in all three types of the eutherian placenta, the endotheliochorial (e.g. cats and dogs), epitheliochorial (e.g. horses and pigs) and hemochorial (e.g. humans and mice). They are associated with invasiveness and access to maternal blood flow (Nakaya and Miyazawa, 2015; Roberts et al., 2016). The fact that the protease domain in PEG10 is highly conserved in all eutherian species (Fig. S1) suggests that the protease activity of PEG10 plays a critically important role. Accordingly, the relationship between PEG10 and RTL1 evolved to provide an adequate feto-maternal interface in a placental-type specific manner. Future studies are needed to determine the molecular mechanisms by which *PEG10* and *RTL1* exert a cooperatively beneficial effect on protecting the fetal capillaries via their critical involvement in the immune tolerance system during pregnancy.

・What do the dotted lines signify in Figure 3?
->
Response

© 2021. Published by The Company of Biologists under the terms of the Creative Commons Attribution License (https://creativecommons.org/licenses/by/4.0/).

The dotted lines indicate the borders between the maternal decidua (de) and spongiotrophoblast (sp), and between the spongiotrophoblast (sp) and labyrinth (lab) layers. We added this explanation in the Figure legend (Line 458-).

Reviewer 2 Advance Summary and Potential Significance to Field...

In this manuscript Shiura et al., investigate the role of the PEG10 viral aspartic protease domain by using CRISPR-cas9 mediated genome engineering in vivo and show, using subsequent analyses on embryonic and placental growth and function, that these modifications are not embryonic lethal unlike Peg10 null embryos as previously shown. Peg10-ASG mice appear to have later defects in fetal vasculature which the authors suggest causes perinatal lethality. These results shedding light on the PEG10 viral aspartic protease domain and the parallels drawn with Rtl1 are intriguing although I have some concerns about the data presented in its current format.
->
Response
We thank reviewer 2 for the favorable comments on our manuscript and have addressed each of the comments in the point-by-point response provided below. By means of these modifications made based on reviewer 2's suggestions, we believe that the data have been made more robust.

Major points:
1. Throughout the manuscript, all defects should be robustly quantified where possible. At present, only one representative image is shown per experiment in Fig. 2 and 3. The phenotype does appear to be quite striking from the images shown but it is currently unclear how representative these are or if there is a spectrum of these defects. For example, is the vastly increased number of CD45 positive leukocytes detected in Fig.3B consistent between +/ASG placentas from different litters? This should be addressed throughout the manuscript because at present I feel the study is descriptive and not as robust as it could be. The appropriate statistics should be used for each analysis. The number of litters, embryos and placentas analysed for each experiment should also be mentioned.
->
Response
In addition to the representative images as shown in Fig. 2 and 3, we have added images of all of the samples recovered from the three litters in the supplementary figures (Figs. S5 and S7). We counted the number of CD45 positive leukocytes in the labyrinth (a total of 21 placentas from four litters were analyzed), and confirmed a significant increase in the *Peg10*-ASG labyrinth (Fig. S7B). In addition, the number of litters, embryos and placentas analyzed for each experiment was cite along with the statistical assessment (analysis of the embryo and placental weight (Figs.1D and E), the *Peg10* expression level in the 12.5 dpc placenta (Fig. S4) and the size of the placental layers (Fig. S6)).

2. The authors should include confirmation that the CRISPR mutation was present as expected in the Peg10-ASG line such as Sanger sequencing of the genomic region etc. Was Peg10 gene expression disrupted or reduced?
->
Response
We added the results of genotyping by sanger sequencing (Fig. S2) and the analysis of the *Peg10* expression level in the placenta, thus demonstrating that its expression was unaffected in the ASG mutants (Fig. S4).

3. From the numbers given in Table 1 (e.g 14% mutant pups versus the expected 50% at weaning) the authors should clarify the chi squared ratios are indeed significantly deviating from expected in the text or table legend for each stage. The authors should also highlight how many litters were used for each experiment. At 12.5 dpc, the numbers of +/+ versus +/ASG embryos (25 versus 16 respectively) was similar to that at 18.5 dpc (28 versus 16 respectively) where the authors suggest the largest effect is taking place, albeit not with the same death rate as at 18.5 dpc. What might be the reason for the low number of +/ASG embryos at 12.5dpc? These numbers suggest something may indeed be occurring at 12.5 dpc, despite their comment that the mutant embryos and placentas exhibited an apparently normal appearance at 12.5 dpc (Fig. S2). The +/ASG placentas already weigh significantly less at this timepoint.
->

© 2021. Published by The Company of Biologists under the terms of the Creative Commons Attribution License (https://creativecommons.org/licenses/by/4.0/).

Response

We added the data and conducted a chi-square test. The modified Table 1 is below.

Table 1. The number of embryos or pups from +/+ dams crossed with +/ASG or ASG/+ sires.

	Litters	Observed +/+ : +/ASG	Expected ratio (1 : 1)	χ^2 value	*P*-value
**12.5 dpc**	8	40 (5) : 29 (1)	34.5 : 34.5	1.75	NS (0.19)
**13.5 dpc**	6	28 (2) : 30 (0)	29 : 29	0.07	NS (0.79)
**15.5 dpc**	12	41 (3) : 55 (3)	48 : 48	2.04	NS (0.15)
**18.5 dpc**	12	**56** (1) **: 22** (**22**)	39 : 39	14.8	**< 0.0005**
**Weaning stage**	9	**30 : 5**	17.5 : 17.5	17.6	**< 0.0001**

The Chi-square test was used to determine whether the difference between an observed and expected frequency distribution (mendelian ratio; 1:1) was statistically significant. NS: Not significant. The number of dead embryos is indicated in parentheses in the "Observed" column.

Although a lower number of +/ASG embryos at 12.5 dpc is still shown in Table 1, a significant difference between the observed and expected ratio was confirmed only at 18.5 dpc and later. Therefore, it is now clearly evident that the effect of the ASG mutation is restricted to the perinatal stage.

4. Is PEG10 protein localisation altered in ASG placentas? Currently staining is only shown for wild type embryos.

->

Response

We added the Peg10 immunofluorescence data for the ASG placentas and confirmed that the localization of the PEG10 protein remained unchanged in the mutant placentas (Fig. S8).

5. Further discussion about the potential mechanism of the PEG10 protease domain role in placental development should be included.

->

Response

As described in the response to reviewer 1's comments, we consider the possibility that the Peg10 protease is involved in the immune tolerance of the mother to the embryo antigens and discuss a potential mechanism in the text (Line 207 to 224, described above).

Minor points:

There is a spelling mistake 'CRIPSR-Cas9' in the first paragraph of the results. Should read CRISPR-Cas9.

->

Response

We have modified the term, 'CRIPSR-Cas9' to 'CRISPR-Cas9' (Line 102).

Fig.3a It may be useful to highlight the labyrinth, junctional zone, decidua regions on the images. Do these regions differ in size?

->

Response

We have drawn dotted lines to indicate the labyrinth layer, spongiotrophoblast layer (junctional zone) and decidua regions on the images. Additionally, we have added the data related to the labyrinth and spongiotrophoblast layer size (Fig. S6). The analysis indicates that only the labyrinth layers displayed a significant decrease in size compared to that of +/+.

© 2021. Published by The Company of Biologists under the terms of the Creative Commons Attribution License (https://creativecommons.org/licenses/by/4.0/).

Fig.3A. Highlight enlarged trophoblast nuclei as mentioned in text.
->
Response
In the original version, we wrote that “while the remaining area stained in bright pink contained enlarged trophoblast cell nuclei”. However, the description “enlarged trophoblast cell nuclei” was not appropriate. Therefore, we replaced the sentence with “while the remaining area stained in bright pink contained the **large nuclei of s-TGC cells**”. In addition, in this part, the existence of s-TGC in the bright pink region is not relevant to the explanation of the fibrinoid necrosis-like staining pattern. Therefore, we have not highlighted the s-TGC in the figure, but rather, totally modified the sentences in this part as follows:

Line 155:
Next, placental morphology was assayed by Hematoxylin and Eosin staining (Fig. 3A; Fig. S6; Fig. S7A). We found that, in addition to a significant reduction in the labyrinth size (Fig. S6), the areas deeply stained in shades of pink-red containing considerable endothelial cell nuclear debris were distributed throughout the labyrinth layer in the dead mutants, and were also observed to a certain extent in the living mutants (Fig. 3A; Fig. S7A). This staining pattern showing a deposition of deep pink pigment around the damaged capillaries is characteristically seen in one of the necrotic forms of cell death, fibrinoid necrosis.

Fig.3A, 3B All analyses should be quantified in some capacity as mentioned in major point 1
->
Response
According to the request, we added HE staining data and demonstrated that the fibrinoid necrosis-like staining pattern was observed in all four of the dead mutants from the three litters and also in certain areas of the two living mutant labyrinth layers from litter C (Fig. S7A). In addition, quantification of the CD45 positive leukocytes revealed that the number of leukocytes in the labyrinth was significantly increased in the *Peg10*-ASG labyrinth (Fig. S7B). A total of 21 placentas from four litters were analyzed and all of the images analyzed are shown in Fig. S7B.

Fig. S3. It might be useful to label cells of interest like the giant sinusoidal trophoblast giant cells etc. on the images.
->
Response
According to the suggestion of reviewer 2, we added asterisks the sTGCs in Fig. S3 (= Fig. S9 in this revised version).

Spelling mistake - VCTASHIELD-Hardset
->
Response
We have modified the mistake, ‘VCTASHIELD-Hardset’ to ‘VECTASHIELD-Hardset’ (Line 311).

Second decision letter

MS ID#: DEVELOP/2021/199564

MS TITLE: PEG10 viral aspartic protease domain is essential for the maintenance of fetal capillary structure in the mouse placenta

AUTHORS: Hirosuke Shiura, Ryuichi Ono, Saori Tachibana, Takashi Kohda, Tomoko Kaneko-Ishino, and Fumitoshi Ishino
ARTICLE TYPE: Research Report

I am happy to tell you that your manuscript has been accepted for publication in Development, pending our standard ethics checks.

© 2021. Published by The Company of Biologists under the terms of the Creative Commons Attribution License (https://creativecommons.org/licenses/by/4.0/).

<u>Reviewer 2</u>

Advance summary and potential significance to field

The manuscript is now much more robust and the authors have addressed my initial concerns. I would like to congratulate the authors on a nice manuscript and a very interesting contribution to the field. It certainly opens other avenues of future research.

Comments for the author

I am satisfied with the changes made and the addition of the substantial amount of new supplemental data which supports their conclusions. The manuscript is suitable for publication.

© 2021. Published by The Company of Biologists under the terms of the Creative Commons Attribution License (https://creativecommons.org/licenses/by/4.0/).
